# Case Report: Spinal Stabilization Surgery Using a Novel Custom-Made Titanium Fixation System for the Spinal Instability Caused by Vertebral Malformation in a Dog

**DOI:** 10.3389/fvets.2021.755572

**Published:** 2021-11-10

**Authors:** Shintaro Kimura, Kohei Nakata, Yukiko Nakano, Yuta Nozue, Naoyuki Konno, Taku Sugawara, Sadatoshi Maeda, Hiroaki Kamishina

**Affiliations:** ^1^The United Graduate School of Veterinary Sciences, Gifu University, Gifu, Japan; ^2^The Animal Medical Center of Gifu University, Gifu University, Gifu, Japan; ^3^Konno 3D Design, Akita, Japan; ^4^Department of Spinal Surgery, Akita Cerebrospinal and Cardiovascular Center, Akita, Japan

**Keywords:** dog, spinal stabilization, surgical treatment, custom-made implant, vertebral malformation

## Abstract

A 2-year-old Maltese was presented with wobbly gait of the pelvic limbs. Based on imaging examinations, a diagnosis of congenital malformation at T5–T8 and severe kyphosis causing spinal cord compression at T6–T7 was made. Dorsal laminectomy and stabilization of T6 and T7 vertebrae were performed. As the size of the vertebrae was small and they were severely deformed, novel custom-made titanium implants were used for spinal stabilization. Clinical signs were resolved 2 weeks after surgery. Although radiographic examination 373 days after surgery showed slight loosening of implants, post-operative course remained uneventful. This report describes the use of novel custom-made titanium implants for spinal fixation surgery in a dog.

## Introduction

Congenital vertebral malformations are developmental abnormalities of the vertebrae that occur frequently in the thoracic column (1, 2). These vertebral malformations may be caused by congenital absence of vertebral vascularization, genetic defects, or teratogenic insult to active cartilaginous proliferation (2–4). Spinal deformities, such as kyphosis, lordosis, and scoliosis associated with vertebral anomalies, tend to worsen during the growth period, and cause vertebral canal stenosis and spinal instability, which injure the spinal cord due to static or dynamic compression (5–8). Surgical treatment is recommended if the case shows neurological signs, such as pain, ataxia, paresis, and/or paralysis, that reduce quality of life (9, 10).

Surgical treatments are aimed at decompressing the spinal cord and stabilizing the affected vertebrae. Several techniques for vertebral fixation were reported such as fixation surgery using pins or screws combined with polymethylmethacrylate or titanium plates (10–12). Although these surgical techniques provide rigid intervertebral fixation and favorable outcomes in most cases, they have potential disadvantages. Advanced skills are required in order to reduce the risks of iatrogenic injury to the vasculature, nerve roots, and spinal cord (13, 14). Moreover, the screw fixation system, which is widely used in veterinary medicine, has been associated with a high incidence of technical failure such as screw pull out and cortical perforation of the pedicles (15, 16). In addition, accurate implant placement may be challenging if there are various malformations with angular deformity in the vertebrae (8). In order to overcome these problems, a novel custom-made implant was developed in human medicine. The patented fixation system is called the SPINE IMMOBILIZATION TOOL (JP2012-136665, PCT/JP2012/065765, WO2012176812 A1) and is used in clinical cases under the approval of Ministry of Health, Labor and Welfare of Japan (17). Herein, we describe a case with deformed thoracic vertebrae that received fixation surgery using novel custom-made spinal stabilization implants.

## Case Presentation

A 2-year-old male Maltese weighing 2.78 kg was presented to the referring hospital (Day 1). The dog had a 2-month history of wobbly gait of the pelvic limbs. A neurological examination revealed ataxia of the pelvic limbs and delayed postural reactions in the pelvic limbs. No abnormality was noted on spinal reflexes. The dog's mental status was alert and superficial pain perception was intact. The severity of the neurological dysfunction was a grade (G) 2 moderate based on grading score from G0 to G5, in which G0—no neurological dysfunction, G1—thoracolumbar pain but no paresis, G2—ambulatory paraparesis (classified as mild, moderate, and severe), G3—non-ambulatory paraparesis, G4—paraplegia with intact deep pain perception, G5—paraplegia with loss of pain perception (18, 19).

**Figure 1:**
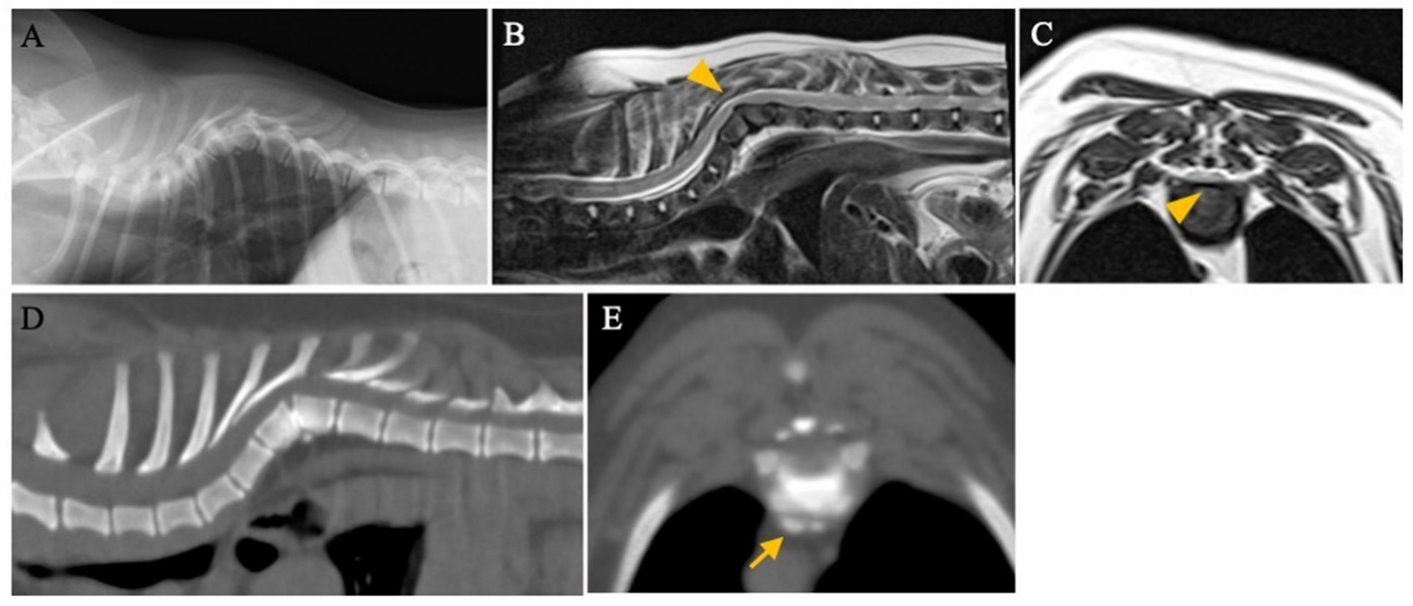
Radiographic, computed tomography (CT), and magnetic resonance images of a 2-year-old dog with wobbly gait of the pelvic limbs. **(A)** Left-right lateral view. There was ventral hypoplasia of T6 and T7 with severe kyphosis. T2-weighted sagittal **(B)** and transverse **(C)** magnetic resonance images at the T6–T7 level. The spinal cord was severely compressed by the deformed vertebrae (arrowhead). **(D,E)** CT images showed sclerotic changes at the vertebral endplates of T6-T7 and the spondylosis deformans at the level of the T6–T7 disc (arrow).

Complete blood cell counts and blood biochemical examination were within normal limits. A radiographical examination revealed severe malformations and malalignment of the vertebrae at T5–T8 (Figure 1A). Ventral hypoplasia of the T6 and T7 vertebral body with kyphosis was identified and the degree of the kyphosis was 76.5° assessed by the modified Cobb method (3, 20, 21). The patient was treated with oral administration of 0.5 mg/kg prednisolone for a week. Despite medical treatment, no improvement in the patient's neurological signs was observed.

On day 7, magnetic resonance imaging (MRI; 1.5-T, MAGNETOM ESSENZA, SIEMENS Healthineers, Tokyo, Japan) of the thoracic spinal cord was performed at the referring hospital. T2-weighted images revealed that the spinal cord was severely compressed by the deformed vertebrae at T6–T7 (Figures 1B,C). On day 22, computed tomography (CT; ECLOS, Hitachi Ltd., Tokyo, Japan) was performed to obtain more detailed information on vertebral malformations and to make a surgical plan at the Animal Medical Center of Gifu University. CT showed ventral hypoplasia of the T6 and T7 vertebral bodies with kyphosis. The vertebral endplates around T6–T7 appeared sclerotic [mean Hounsfield Units (22): 1089], and osteophytes were observed in the same area (Figures 1D,E), suggesting spinal instability (23, 24). No other malformations of the vertebrae and spinal cord lesions were observed. Based on the MRI and CT findings, a diagnosis of hemivertebrae of T6 and T7 with kyphosis and possible spinal instability causing spinal cord compression was made. Dorsal laminectomy to decompress the spinal cord and spinal fixation surgery were planned. The size of the thoracic vertebrae was small (height, width, length of T6 and T7 were 7.3, 6.4, 8.4 mm; and 7.4, 6.1, 9.4 mm, respectively) and the vertebrae were severely deformed. Accurate placement of the implants was thought to be difficult; therefore, custom-made spinal stabilization implants were used.

**Figure 2:**
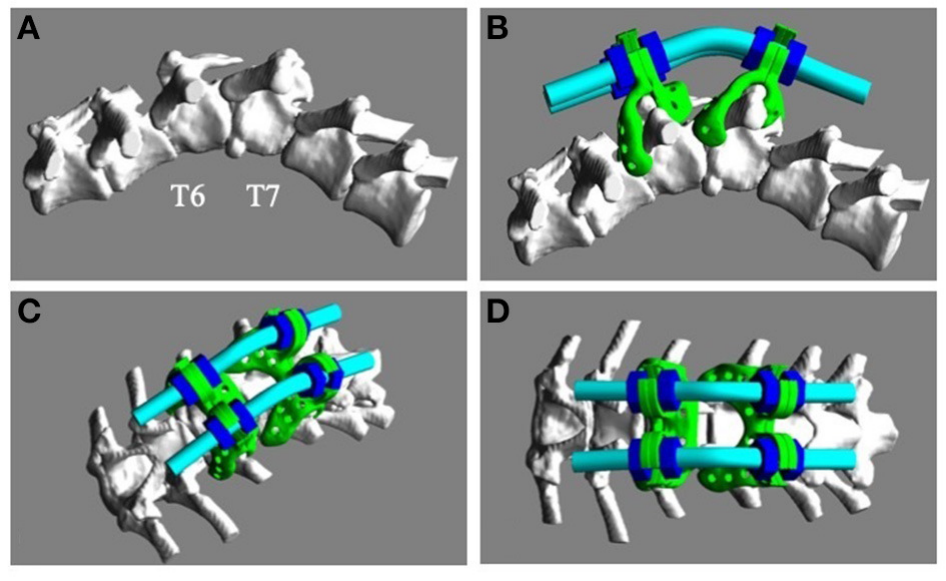
Design of the custom-made titanium implants. **(A)** The deformed vertebrae were realigned to a suitable anatomical relationship using 3D modeling software. **(B–D)** The titanium implants consisted of spinal covers (green), fixing rods (cyan), and nuts (blue).

The bone data was extracted from the pre-operative CT data and transferred to 3D modeling software (Freeform; 3D Systems Inc., SC, USA). The deformed vertebrae were realigned to a suitable position (Figure 2A). The custom-made titanium spinal stabilization implants (“the spinal covers”) consisted of a 1.5 mm-thick titanium plate, which was made of grade 23 titanium Ti6Al4V powder (Arcam AB, Molndal, Sweden) containing O_2_ < 0.13% as the printing material, and had 1-mm pores for bone invasion. The titanium rods (Aoyama Seiko Co. Ltd., Shizuoka, Japan) with a diameter of 3.0 mm were bent in advance to allow for placement on the spinal covers (Figures 2B–D).

**Figure 3:**
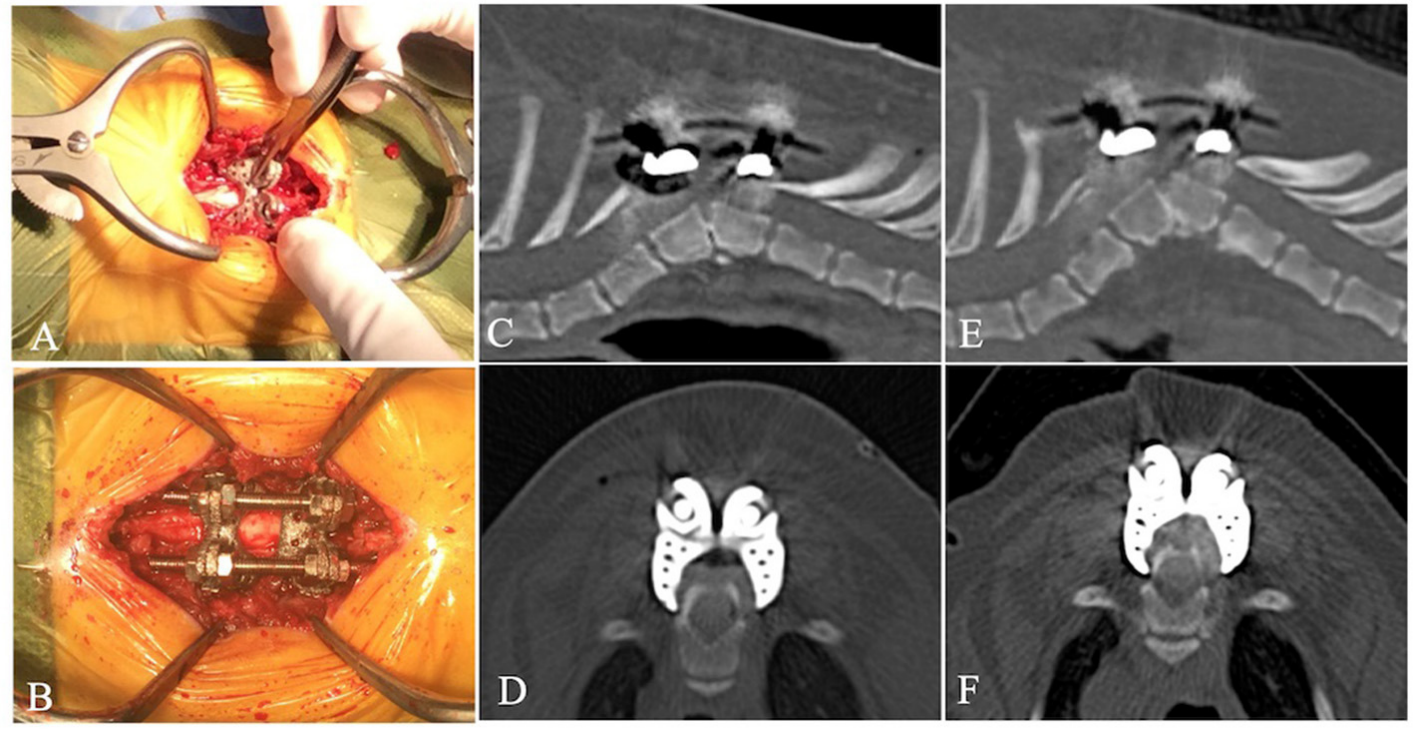
During surgery, tweezers were used to mount the spinal covers to the lamina and the heads of the ribs and enclose the spinous process **(A)**. The rods were bridged between the spinal covers and locked by the nuts **(B)**. Post-operative CT images immediately after surgery showed the degree of kyphosis [**(C)**, left-right lateral view; **(D)**, ventral-dorsal view]. Post-operative CT images at 372 days after surgery showed no bending or breakage of the implants and no collapse of the adjacent intervertebral space. However, slight loosening of the implants was observed and the ventral side of the T7 vertebral body was slightly collapsed. There was bone regeneration around the implants [**(E)**, left-right lateral view; **(F)**, ventral-dorsal view].

On day 69, dorsal laminectomy and fixation of the T6 and T7 vertebrae were performed. Surgical procedures were performed with informed consent from the owner and in accordance with guidelines regulating animal use and ethics at Gifu University. The dog was anesthetized with propofol (PropoFlo 28; Zoetis Japan, Tokyo, Japan) and maintained by isoflurane (Isoflurane for Animals; Intervet, Tokyo, Japan). Fentanyl (3–15 μg/kg/h) and ketamine (0.12–0.6 mg/kg/h) were administered during surgery. For dorsal laminectomy, the spinous processes of T5 and T6 were completely excised and the articular processes were preserved. After excision of the dorsal lamina, cerebrospinal fluid pulsation was confirmed. The spinal cover was designed to cover the surface of the lamina and the heads of the ribs. To better fit the implants to the spine, the musculature and connective tissues around the spinous process and vertebral arch of T6 and T7 were removed. Each spinal cover had hooks that anchored the cranial or caudal edge of the lamina and the heads of the ribs. A pair of spinal covers was fixed by tweezers to pinch a dorsal lamina. The implants were firmly attached to the vertebrae by anchoring the hook to the dorsal lamina and the head of the ribs of T6. Another pair of spinal covers was attached to the vertebrae of T7 in the same way. Subsequently, the fixing rods were bridged between the rod holes of the spinal covers of T6 and T7, and locked by the titanium nuts, which allowed for traction and slight modification of the vertebral alignment (Aoyama Seiko Co. Ltd.) (Figures 3A,B). Finally, dorsal spinal fascia were apposed over the implants and the subcutaneous tissue layers were closed in a routine pattern. For post-operative analgesia, fentanyl (2 μg/kg/h) and ketamine (0.08 mg/kg/h) were administered. The degree of kyphosis was improved to 62.7° (Figures 3C,D), assessed by the modified Cobb method, compared to its pre-operative level. Immediately after surgery, decreased conscious proprioception of the pelvic limbs was noticed and the severity of neurological dysfunction deteriorated to G2 severe. Postural reactions were gradually recovered and the severity of dysfunction improved G2 mild 2 weeks after surgery.

On day 441, CT showed no bending or breakage of the implants and hyperostosis was observed around the implants (Figures 3E,F). However, the distance between the implant and the floor of the vertebral canal was shortened (3.7 mm at caudal side of the T6 vertebra and 3.8 mm at the cranial side of the T7 vertebra), compared to immediately after surgery (4.9 mm at caudal side of the T6 vertebra and 4.7 mm at the cranial side of the T7 vertebra). The Cobb angle was 68.1°, which was slightly steeper compared to its angle immediately after surgery. Although there was no interspace collapse of the adjacent intervertebral space, the intervertebral space between the T6 and T7 vertebrae was slightly reduced. Clinically, the gait, postural reactions, and spinal reflexes of the four limbs were normal, and the severity of neurological dysfunction was improved to G0.

## Discussion

Congenital vertebral malformations often result in vertebral canal stenosis and spinal instability. Vertebral malformation and malalignment tend to worsen during skeletal growth, and the spinal instability may be progressive and result in spinal cord injury at any age (5–7). In humans, kyphoscoliosis is associated with spinal instability and causes dynamic spinal cord compression (6, 7, 25). Decompression of the spinal cord and/or stabilization of vertebrae are recommended (21, 26). In the veterinary field, vertebral stabilization for severe angular malformations is effective (8). In the present case, the patient had severe vertebral anomalies and sclerotic changes of the endplates and spondylosis deformans, which suggested progression of the deformity (27).

For the surgical treatment of spinal instability, screws or pins with polymethylmethacrylate, or titanium plates were frequently described (10–12, 28–30). These techniques provide sufficient fixation, but have risks of iatrogenic injury to nerve roots or the vertebral artery because of the misplacement of implants (11, 12, 28). In humans, the rates of screw misplacement for the free-hand technique were between 28 and 43% in cases of vertebral malformations such as scoliosis (31, 32). Thus, the accurate insertion of implants can be challenging when there is severe angular deformity of the vertebrae. Anatomical variations of the vertebrae among different dog breeds further increase the risks of complications associated with iatrogenic injury (8, 14). Our custom-made spinal cover only requires mounting of the implants over the spinous process and the lamina; thus, insertion of pins or screws in the pedicles or vertebral bodies were not performed. When placing the implants, we need to almost completely remove soft tissues from the vertebrae, which may interfere with the vascularization of the vertebrae. By limiting the area of coverage to the lamina and the heads of the ribs, we were able to minimize the extent of soft tissue removal. This system allows intraoperative risks of injury to important structures to be avoided. In addition, this system requires only a simple procedure and no special equipment, such as a fluoroscope or navigation system, is used. In the present case, there were no intra- and post-operative complications.

The custom-made spinal cover consisted of a patient-specific spinal cover and fixing rods. A previous study found the durability of these implants were equal to or higher than that of conventional titanium implants (17). The patient-specific spinal cover was made based on a 3D model of the patient's vertebrae. This structure increases area of contact between bones and implants, which allows for rigid fixability. In the ossification process, rigid fixation promotes bone fusion rate (33). This spinal cover has 1-mm pores for bone invasion, which allow for long-term fixation through ossification (17). In the present case, postural reactions decreased a few days after surgery, but recovered 2 weeks after surgery and neurological symptoms improved 3 months after surgery. CT on day 441 showed the reduction in distance between the implant and the floor of the vertebral canal, and the intervertebral collapse between T6 and T7, leading to an increase in Cobb angle. These changes are likely to be associated with the connection loosening between implants and bone. The lack of contact between the bone and implant may have resulted in instability of implants.

Since there was no acute worsening of neurological signs and the dog remained in a good condition 12 months after surgery, the changes might have progressed gradually over time. To avoid these changes and to increase the rigidity of the stabilization, the area covered by the implants could be modified. In our case, the implant was mounted on the lamina and the heads of the ribs. The heads of ribs are mobility, which could result in an increased risk of dislodging the implant device. Further studies are needed to determine the area covered by the implant. Furthermore, the multi-vertebral fixation could be useful. The method allows for an increase in the support range of the implant and the wider distribution of forces on the vertebral bodies (34). However, it should also be noted that multi-vertebral fixation may increase the risk of adjacent segment disease (35). The combination of wire fusion might also be effective (36).

In the present study, we reported the use of a novel vertebral fixation system for spinal instability due to vertebral malformations. Although long-term follow-up is necessary, this system allows for safe and effective stabilization of vertebrae.

## Data Availability Statement

The original contributions presented in the study are included in the article/supplementary material, further inquiries can be directed to the corresponding author.

## Author Contributions

SK, KN, YNa, YNo, SM, and HK contributed to conception and design of this report. NK and TS contributed to design the implants. SK wrote the first draft of the manuscript. All authors contributed to manuscript revision and read and approved the submitted version.

## Conflict of Interest

The authors declare that the research was conducted in the absence of any commercial or financial relationships that could be construed as a potential conflict of interest.

## Publisher's Note

All claims expressed in this article are solely those of the authors and do not necessarily represent those of their affiliated organizations, or those of the publisher, the editors and the reviewers. Any product that may be evaluated in this article, or claim that may be made by its manufacturer, is not guaranteed or endorsed by the publisher.
